# Brexit: A Boon or a Curse for Animals Used in Scientific Procedures?

**DOI:** 10.3390/ani11061547

**Published:** 2021-05-25

**Authors:** Rachel Dunn

**Affiliations:** School of Law, Northumbria University, Newcastle upon Tyne NE1 8ST, UK; rachel2.dunn@northumbria.ac.uk

**Keywords:** animal experimentation, animal (scientific procedures) Act 1986, Brexit, animal welfare

## Abstract

**Simple Summary:**

Many animal welfare laws, including those regulating animals used in scientific procedures, have been impacted by the EU. The Animals (Scientific Procedures) Act 1986 (ASPA) is the main legislation in the UK governing animals used in scientific procedures and was amended with the introduction of Directive 2010/63/EU. Though in some ways it seemed that the Directive would improve the welfare of animals in scientific procedures, this may have not been the case. In light of Brexit, the UK faces challenges in maintaining the protection for these animals, particularly with no longer having access to REACH and the risk of duplicate experiments. There are also, however, opportunities to increase the protection already established and build upon the UK’s reputation of being a leader in animal welfare. This includes more transparency of experiments carried out, utilizing more non-animal methodologies and ending severe suffering. This article will explore the potential implications of Brexit on the welfare of animals used in experiments, focusing on legislation surrounding their welfare and protection. It will also suggest ways in which this protection can be progressed, with potentially more freedom to amend or introduce legislation to do so.

**Abstract:**

The UK has long been hailed as one of the world leaders in animal welfare. Within the UK, animals used in experiments are provided some protection under the Animals (Scientific Procedures) Act 1986 (ASPA). This Act was impacted by European Union (EU) Directive 2010/63/EU, and subsequently the ASPA was updated to reflect any changes required. While the Directive is very similar to the protection the UK already afforded to animals used in experiments, there were some advances that the Directive provided that were not present in the ASPA. On paper, the changes introduced were promising but may not have been achieved in practice. In 2016, the British public voted to leave the EU, which presented concerns over animal welfare protection and legislation provided by EU law. With the completion of Brexit, there may be an opportunity to diverge from the Directive to advance protection for animals used in experiments. This article explores the influence that the EU has had on animal experimentation in the UK, the potential implications of Brexit on the welfare of animals used in experiments and suggests ways in which this protection can be progressed, with potentially more freedom to amend or introduce legislation to do so.

## 1. Introduction

Animal experimentation has a long history in the UK and the first legislation to prohibit painful experiments on animals was passed in 1876 [1]. The legislation which now governs animals used in experiments is the Animals (Scientific Procedures) Act 1986 (ASPA). This was amended in December 2013, to transpose Directive 2010/63/EU (the “Directive”), which aimed to update and replace Directive 86/609/EEC, creating more protection for the animals used in procedures and further promote the 3Rs (replacement, reduction and refinement). In 2019, 3.4 million procedures were carried out under the ASPA in Great Britain, the lowest annual amount since 2007 [2], though this was still the highest number of any Member State in the European Union (EU). When the Directive was introduced, it was acknowledged that the protection provided by the UK in the ASPA was already similar and that there would be little difference to the operation of it [3]. Thus, there was little change within the UK, but some elements already found in the UK’s domestic legislation were strengthened or further enshrined.

In 2016, the British people voted to leave the EU through a referendum, starting a complex process of separating from a legal system of which the UK had been a Member State since 1973. Primarily established as the European Coal and Steel Community in 1951, the aim was to create a common market in coal and steel. Since then, it has developed into the EU, often described as an emerging superpower [4]. The different institutions of the EU are the European Council, the Council of the EU, the European Commission, the European Parliament and the Court of Justice of the EU. The EU creates laws, such as Directives and Regulations, which become law in each of the Member States. Further, Member States are governed by the four freedoms that make up the Single Market of the EU: the freedom of movement of goods, persons, services and capital. The UK’s legal system has become entwined with that of the EU and many domestic laws have been passed to comply with EU law. There was no ability for Parliament to replace all of these laws prior to the withdrawal from the EU [5], due to the quantity and complexity. For example, more than 80% of current UK animal welfare legislation comes from the EU, with over 40 pieces of law, of which 8 relate to animals used in experiments [6]. There were concerns that Brexit would cause huge gaps in our domestic legislation if EU law was not retained. To avoid this the European Union (Withdrawal) Act 2018 was passed, which retains the majority of EU law, taking a ‘snapshot’ of existing legislation on exit day, converting it into domestic legislation [7]. Thus, the Directive will not disappear from the UK’s domestic legislation now that the UK have left the EU, as it is retained EU-derived domestic legislation under the European Union (Withdrawal) Act 2018.

There is now an opportunity, however, to review the current legislative framework governing animals in experiments and determine what is working as intended and where the UK can strengthen the law without the constraints from EU membership. This is particularly important when considering that the UK is often claimed to be a “world leader” in animal welfare, due to being the first country in the world to pass animal welfare laws and pushing for change within the EU. Various UK Governments have reiterated this over many years in various policy documents and speeches. It was most recently stated in the Government’s ‘flagship action plan’, Action Plan for Animal Welfare, published May 2021, outlining the legislation they plan to implement over the coming years. It is important to note that the action plan does not include any aims for animals used in experiments, and they have been left out of the manifesto, but highlights how the Government wish to use Brexit as an avenue to strengthen various animal welfare laws. It is stated that the ‘departure from the EU has provided us with an opportunity to do things better’ and that they wish to ‘solidify and enhance our position as global leaders’ [8]. This is promising, as there are concerns that the UK are now trailing behind other countries for animal welfare. For example, various other countries have had harsher sentences for animal welfare offences for some time [9], something long campaigned for in the UK, and the Animal Welfare (Sentencing) Act 2021 has just been passed to implement this. The lack of provisions and protection for some animals has recently led to the UK being dropped in the Animal Protection Index from A to B overall [10]. Comparing the results from other countries who scored a B overall, namely Sweden, the Netherlands and Austria, the UK are falling short of their standards in many categories, but particularly for animals used in scientific research. The other countries named all scored A in this category and the UK scored C [11]. Thus, it is clear that to maintain the reputation as a world leader in animal welfare and protection and to advance in position in the Animal Protection Index, there is more the UK can be doing.

It is acknowledged that there are many arguments in the literature as to the necessity or otherwise of animal experimentation. For example, Harrmann and Jayne argue that the ‘industry wastes intellectual, scientific, and financial resources and causes harms not just to animals but also to humans’ [12] (p. xxxvi). In contrast, there are arguments that in order to make progress in medicine and biology, the use of animals is necessary, with activists in antivivisection engaging with criminal activities and spreading misinformation which is harmful to the progress [13] (pp. xviii-xx). It is not the purpose of this article to engage with such debates, as important as they are, but is instead a discussion of the law relating to animals used in experiments and whether this law can be improved now that the UK has left the EU. It will highlight the issues with the law and where it has not been working as intended in practice, but will not determine or draw conclusions as to whether experimentation on animals should be abolished.

Animal welfare in experimentation is a vast and complex subject and it is not possible to cover all aspects of animal experimentation and the relevant law in this article. Thus, this article will focus on several important aspects of the ASPA that have been highlighted by academics and practitioners as the most important. It will discuss the licensing procedure under the ASPA, specifically the severity limits and the harm–benefit test, the lack of transparency of research involving animal experiments, and the promotion of the 3Rs and slow development of alternative methods of testing. Section 2 of this article critiques the law as it stands, demonstrating where the Directive has attempted to advance the UK’s already established legislative regime. Section 3 of this article analyses the impact of EU membership on animals used in experiments and where the UK has benefitted or been restricted by this membership. This includes a discussion of Regulation (EC) No. 1907/2006 concerning the Regulation, Evaluation, Authorisation and Restriction of Chemicals (REACH). The final section explores what the UK can do in the post-Brexit world to improve the welfare of animals used in experiments. Ultimately, this article concludes that Brexit in the short term will be a curse for animals used in experiments but has great potential to create positive outcomes in the long term.

## 2. Background to Animal Experimentation and the ASPA

The ASPA is the legislation regulating experiments or other scientific procedures on living animals in the UK. It has been amended since its enactment, particularly with the introduction of the Directive, which was largely based on the ASPA and contained many of the qualities praised in the UK legislation. For example, the ASPA already contained provisions for seeking alternative non-animal models, though the Directive further enshrined the principles of the 3Rs into legislation. There were already provisions for licensing, severity categories and inspections, though the Directive brought about some changes to these processes. Due to this, instead of passing new legislation, the Government just amended the ASPA to reflect any changes to be transposed by the Directive which were not already present in the domestic legislation. An unusual aspect of the Directive is Article 2, which prohibited Member States from increasing the protection provided under the Directive domestically, although they could retain any greater welfare measures in force before November 2012. This means, as non-governmental organizations (NGOs) have recognized, the UK’s ability to increase the welfare, and potentially restrict further animals used in experiments, has been limited [14]. This section highlights the important sections of the ASPA and the Directive and outlines the strengths and weaknesses of each section.

### 2.1. Licensing

Before analyzing the strengths and weaknesses of the legislative framework, the procedure for carrying out experiments on animals must be outlined. All scientific procedures carried out on animals are required to have a license approved by the Home Secretary. The ASPA applies to protected animals, defined in s.1 as ‘any living vertebrate other than man’. A regulated procedure is defined under s.2 as ‘an experiment or other scientific procedure applied to a protected animal which may have the effect of causing that animal pain, suffering or lasting harm.’ What is important about the ASPA is that it legalizes practices which would be criminal offences under other legislation, such as the Animal Welfare Act 2006, which explicitly exempts under s.58(1) acts lawfully done under the ASPA. The granting of a license falls with the Home Office, and the Secretary of State holds the regulatory power when granting a project license. They must be satisfied that the evaluation of the program of work is favorable, evaluating certain criteria is met under s5B(3), such as that the objectives of the program and its predicted scientific benefits or educational value and the compliance of the program with the 3R principles. The Secretary of State is supported by the Animals in Science Committee (ASC), established to comply with the Directive, providing independent advice on issues relating to the ASPA.

The ASPA requires two separate licenses to be granted before a regulated procedure can be carried out on a protected animal: a personal [15] and a project license [16]. It is important to note that the institution or company where the procedures take place also need to have been granted an establishment license [17]. The majority of establishment licenses in 2019 were granted to universities/medical schools at 53%, who also accounted for 79% of project licenses [2] (p. 20). A personal license must be held by the individuals involved in the procedure and is subject to review by the Secretary of State every 5 years [18]. It specifies the nature of the procedures the holder of the license is authorized to make, the type of animal involved and the place at which the procedure can be carried out. It also requires that the holder at all times acts in a way which is consistent with the 3Rs and must include conditions which are designed to limit the pain, suffering or distress caused to the animals. A project license, which authorizes the procedure itself, includes the type of animal to be used and where the work is to be carried out. The project license must also require that the holder has ensured that the work specified does not involve an application of a regulated procedure for which there is a satisfactory scientific alternative method or testing strategy not entailing the use of a protected animal [19].

### 2.2. Severity Limits/Categories and the Harm–Benefit Assessment

Each procedure is allocated a severity category—the categories have changed since the Directive was transposed. Prior to the Directive, the old ASPA regulatory framework, severity limits were “unclassified”, “mild”, “moderate” and “substantial”. Substantial was defined as: ‘Procedures that have the potential to cause a major departure from an animal’s normal state of health or well-being’ [20] (p. 34). This includes procedures such as major surgical procedures which were expected to cause significant morbidity or mortality. This issue with the previous system of classification of severity limits was the assessment the overall suffering. Under the old framework, each protocol, or individual procedure, was assessed and assigned a severity limit, looking at the ‘worst possible outcome’ for any animal used in the protocol. Once this was done, the overall severity limit, or band, was assessed for the project, which took into account the proportion of animals expected to reach each severity limit, basing the decision on aggregate suffering. Thus, the more animals subjected to a protocol which was assessed as moderate would create an overall severity rating of moderate, even though a small proportion may have been subjected to procedures which amounted to the substantial limit. This means that the severity limit for each project overall was not ‘representative of any individual protocol that subjected animals to the most severe suffering’ [21] (p. 7). Further, the old ASPA also only accounted for assessment prior to the experiment and during the project license application stage. There was no measurement as to whether the suffering that had been granted under the project license had been met or exceeded. This meant it was very difficult to analyze whether the procedure had gone beyond that which was expected or legally granted and did not provide guidance for future research projects on assessing severity limits to avoid similar issues.

The Directive changed severity limits and their categorization. Prior to the Directive, the ASPA classified the highest suffering as ‘substantial’, which was then replaced by ‘severe’ under Article 15. Schedule 2C, paragraph 23, states that a project license must include a condition that when a procedure has been applied to an animal as part of the specified program, a suitably qualified person classifies the severity of the procedure as “non-recovery”, “mild”, “moderate” or “severe”. Non-recovery procedures are ‘procedures which are performed entirely under general anesthesia from which the animal shall never recover consciousness’. Severe is defined as: ‘Procedures on animals as a result of which the animals are likely to experience severe pain, suffering or distress, or long-lasting moderate pain, suffering or distress as well as procedures, that are likely to cause severe impairment of the well-being or general condition of the animals…’ [22]. From the definition of substantial provided above, it can be seen that the new upper limit from the Directive is much more detailed, taking into account several aspects of welfare. There is a further category of “sub-threshold”, which is a procedure causing less pain than inserting a hypodermic needle according to good veterinary practice. This Article also prohibited procedures involving severe pain, suffering or distress that is likely to be long-lasting and cannot be ameliorated, though there is a ‘safeguard clause’ under Article 55 discussed in further detail below. Article 39 of the Directive provided for retrospective assessments of projects, evaluating whether the objectives of the program were achieved, the harm inflicted on animals and the severity of the procedures and any elements that may contribute to further implementation of the 3Rs. The new identification of the severity limits and retrospective assessments under the Directive allowed for actual harm to be captured after the project, and not just the estimated harm prior. It also allowed for the overall level of suffering to be experienced by every individual animal to be considered, meaning that if the majority or average number of animals will experience moderate suffering but if a single animal may experience severe suffering, it should be placed in the severe category. This also includes cumulative suffering, accounting for a series of events that on their own would not be severe, but when taken together increase the suffering to severe. In the UK in 2019, 91% of all procedures were assessed as sub-threshold, mild or moderate and the rest were non-recovery or severe [2] (p. 6).

Though it seemed the Directive was attempting to specify what would be unacceptable suffering to subject animals in experiments to, it has not really done that in practice and some have taken issue with it. For example, Beauchamp and Morton have argued that, though on paper the upper severity limit seems promising, there is little guidance on how to measure it and it ‘lacks a careful and detailed analysis of what we regard as the two essential dimensions that must be articulated in assessment of severity of harm–namely, the *intensity* and the *duration* of the harm’ [23] (p. 438). Further to this, they highlight how the cumulative suffering resulting from a series of procedures carried out on an animal is difficult to calculate. Their second main issue with the Directive’s severity limit is that the prohibition can be set aside under Article 55(3) (the ‘safeguard’ clause), where there are exceptional and scientifically justifiable reasons. Beauchamp and Morton conclude that there is not an ‘absolutely binding upper limit’ on the severity of pain that can be inflicted [23] (p. 439). While this is true, the EU have reported that no Member State has initiated a safeguard clause since the Directive took effect in January 2013 [24]. The issue with severity limits across Member States, however, is the lack of guidance and definition of these terms, particularly what ‘cannot be ameliorated’ means and the level of suffering that has to take place for it to be ‘severe’ [25]. As there is little guidance, and what does exist is general rather than detailed and specific, there are concerns that there is ‘considerable variation’ as to how severity limits are applied [26].

What has been allowed since January 2013 is a substantial number of procedures to be carried out that were assessed as severe. In 2017 (the most recent statistics), across Member States the number of procedures carried out in the different severity classifications are displayed in Table 1 below:

In the UK in 2019, less than 10% of all scientific procedures carried out were in the severe or non-recovery classifications [2] (p. 6). Though the percentage is low, when taking into account the total number of animals used in scientific procedures, this is still a substantial number of animals subjected to an extreme level of suffering. There is also no way to compare whether the Directive has improved classifications, due to no requirement to assess and report the actual level of suffering experienced by an individual animal prior to the Directive.

When the Directive was introduced, there was much discussion surrounding the severity limit changes to the ASPA. Balls and Hudson argued that ‘substantial’ would have been a better term to use in the Directive and that there needed to be guidance ‘of the highest quality’ to ensure the most humane methods are being used [28] (p. 111). Further, there is belief that severity limits are not being assessed appropriately [29], and this may mean that experiments are erroneously more likely to be placed in the moderate band, rather than severe [30]. There is now guidance being developed to help scientists with severity categories and ensure that they are being placed in the correct category [31].

Prior to the Directive, s.5(4) of the ASPA provided for a harm–benefit assessment to be conducted by the Secretary of State when considering granting a project license, requiring them to weigh the likely adverse effects on the animals used against the likely benefit to accrue as a result of the procedure. This was provided for in the Directive under Article 38, and an amended version appears in s.5B(3)(d) of the ASPA. It is more detailed than prior to the Directive and states the Secretary of State must: ‘carry out a harm-benefit analysis of the programme of work to assess whether the harm that would be caused to protected animals in terms of suffering, pain and distress is justified by the expected outcomes, taking into account ethical consideration and the expected benefit to human beings, animals or the environment.’ This adds a utilitarian aspect to reviewing licenses, attempting to ensure any potential benefits of the results of the experiment outweighs the harm caused. An advantage of the harm–benefit test is that it can be, and has been previously, used to introduce policy bans by not granting a license for particular experiments rather than a ban on certain experiments through legislation. In the UK, for example, decisions not to grant licenses for experiments relating to cosmetics and the use of great apes resulted effectively in a ban of these experiments [14]. It is acknowledged that it can be challenging to determine whether the harm is justified by the benefits and, coupled with how experiments are reviewed and authorized, there may not be harmonization between Member States [26]. Further, only with transparency of research, discussed in more detail below, can we truly know whether the harm–benefit assessment has been carried out properly and with adequate information.

### 2.3. Promotion of the 3Rs

The 3Rs (replacement, reduction and refinement) have long been a staple element of animal experimentation governance and are ‘fundamental to the philosophy underlying the guidelines and legislation that regulate animal experimentation’ [32] (p. 379). The 3Rs originated from Russell and Burch in 1959 as a way to improve the treatment of animals used in experimentation and advance the quality of scientific research [33]. They were very much a ‘foundation for future developments’ for experiments involving animals, working toward new developing new methodologies and non-animal methods [34]. Prior to the Directive, the ASPA did include an element of the 3Rs in s5(5), which stated: ‘The Secretary of State shall not grant a project licence unless he is satisfied the applicant has given adequate consideration to the feasibility of achieving the purpose of the programme to be specified in the licence by means not involving the use of protected animals.’ Thus, there was a clear indication that research should first try to find an alternative to using animals and that they should only be used as a last resort. Though the principles of the 3Rs were present, there was criticism of their actual use, mainly from anti-vivisection groups. For example, in a Select Committee on Animals in Scientific Procedures report in 2002, it was stated: ‘Those who are fundamentally opposed to animal experiments consider that replacement in the only “R” worth arguing for. Refinement and reduction, they argue, merely serve as a smokescreen to allow scientists to experiment with animals’ [35] (p. 37). Further, research shows the lack of reliable data and development of clinical interventions when using animal models [36], with non-animal models able to generate faster and more reliable predictive results for humans [37]. Even with this evidence, the statistics provided above indicate that the use of animal models in experiments is the preferred method and replacement has been slow to integrate into the scientific communities. Taylor is of the opinion that one of the reasons for this is the bureaucracy involved in regulation of experiments and the failure to provide incentives or rewards for using and evaluating new non-animal methods in research [38] (p. 597). Lauwereyns argues that there may be ‘concept fatigue’ with the 3Rs, as they do nothing to resolve debates between those for and against animal research and ‘putting them into practice turns out to be a problematic exercise’ [39] (p. 10). This may be due to conflicting ideas of which R to use, conflicting interpretations of how to apply them and the ethical issues hidden within them [40].

The Directive very much made use of the principles of the 3Rs within its text. Article 4 stipulates that Member States shall ‘where possible’ ensure alternative methods to animals be used that are scientifically satisfactory. Article 47 of the Directive provides that Member States contribute to the development and validation of non-animal methods. Thus, we can see the attempt by the EU to work towards the replacement of animals in experiments, and the 3Rs were more enshrined into the ASPA as a result. S.2A of the ASPA states that the Secretary of State must exercise their functions under the Act with the view to ensuring compliance with the 3Rs, which are then defined in s.2A(2). When granting a project license, the Secretary of State must be satisfied that the program of work complies with the principles of the 3Rs under s.5B(3)(b). It can be concluded, therefore, that the enshrining of the 3Rs into domestic legislation more extensively should have ensured greater compliance with them, but this may not have been the case. For the UK, Calley argues that Article 4 ‘is simply a regurgitation of what went before and the explicit inclusion of the Three Rs is nothing more than an exercise in window dressing’ [21] (p. 20). This, coupled with the fact the Directive did not provide a targeted schedule or timeframe for the reduction in animals used in experiments, means that the advancement of the Directive may be fruitless in practice [3]. It is still potentially too early to tell whether the Directive has been effective in this area, though there is some further discussion in the later sections of this article. In the first report on the effectiveness of the Directive in 2017, 81% of users felt that the person responsible for overseeing welfare and training had been effective in their contribution to the implementation of the 3Rs. While the majority felt that training was being provided, only 30% of respondents felt there had been a positive impact of the Directive on an increased focus on alternatives to animal methodologies. Obstacles to using alternatives included a lack of knowledge of them, non-availability in particular fields and lack of sharing knowledge or advice [41]. There is evidence that relevant stakeholders feel as though the Directive is working toward advancing the 3Rs and had a positive impact, but more research needs to be done to objectively determine whether this is the case.

Though the Directive provides for greater use of the 3Rs, the UK Government does not wholly contribute towards their development. The Government does contribute to the development and validation of non-animal methodologies, but it has been noted that clearer legislative or policy development in the area is needed [14]. Further, it has been highlighted that developments in replacing the use of animals ‘occur more by accident than by design’ [42] (p. 88). Even with the appearance of support for the 3Rs, little attention is given to them by those who regulate animal experimentation. For example, in the Home Office report, which is cited extensively in this article, there is no mention, never mind statistics, demonstrating how the scientific community are attempting to incorporate the 3Rs. It appears that the measurement of the 3Rs and how many procedures on animals were avoided during each year are either of little importance or the methods for measuring this are currently insufficient. The main domestic work for the advancement of the 3Rs is through the work of the National Centre for the 3Rs (NC3Rs), who work with the research community using technology to replace animal studies. Where this is not possible, they support researchers in how best to design experiments to increase animal welfare by robust research and provide guidance on improving animal welfare [43]. The coalition Government in 2014 did attempt to commit to reducing the number of animals used in research and published a Delivery Plan outlining how they will satisfy the commitment [44]. Their plan included developing domestic and international programs focusing on advancing and influencing the adoption of the 3Rs, and a program promoting understanding about the use of animals where there are no alterative non-animal methodologies. The key objectives to achieve this included supporting the NC3Rs in delivering ‘high quality’ programs, facilitating data sharing and collaboration and enhance the role of Home Office inspectors in advancing the 3Rs [44] (p. 20). This was unfortunately not taken further, and the Royal Society for the Prevention of Cruelty to Animals (RSPCA) highlighted that the plans seem to have been ‘forgotten’ urging the current Government to go ahead with it [45]. One of the reasons that this plan may not have been taken forward is the lack of timeframe or targets for reducing the number of animals used, meaning it lost momentum. If revisited, it is imperative that there are clear and achievable goals identified in terms of strategy periods and numerical targets.

There needs to be clear initiatives for the scientific community to fully engage with non-animal methodologies. This is beginning to happen, with some Member States leading the way. For example, the Netherlands National Committee for the protection of animals used for scientific purposes (NCad) released a plan in 2016, the first Member State of the EU to publish a plan of how they will phase out animal experimentation [46]. They highlight how some experiments, such as regulatory safety testing of chemicals and food ingredients, can be phased out by 2025, though regulatory pre-clinical research will not be able to be phased out within this accelerated pace [46]. Their transition strategy involved various recommendations, which included investing in the acceptance of non-animal methods and ensuring better use is made of results from human subjects [46]. Unfortunately, the ambition and innovative plan has not been widely accepted by other Member States and the timeline for phasing out animal research has been criticized, mainly that it risks safety of medical treatment and restricts research [42]. Though criticized, the plan is clearly much more robust and necessarily detailed in order to work toward the goal, compared to the 2014 UK plan, which was quite weak in its approach to achieving the plans set out.

### 2.4. Transparency of Research

An element of animal experimentation legislation is the importance of transparency of findings. This is for two main reasons: to avoid unnecessary duplicate studies and so that the public understand how they are financing animal experimentation [47,48]. In the UK, in order for the public to ensure that experiments are carried out accordingly and that the Home Office has granted appropriate licenses, a Freedom of Information Act 2000 (FOIA) request can be made. This is often used by NGOs and activist groups, and is a useful tool. However, it can be restricted under the FOIA. Under s.44 of the FOIA disclosure of information held by public authorities is prohibited where such disclosure is prohibited by any enactment. This prohibition exists in s.24 of the ASPA, which stops the Home Secretary from releasing any information received in confidence under the ASPA or obtained when discharging functions under the ASPA. This is the case even where the provider has no objection to its disclosure. Essentially, this means that the Home Office does not need to make details of experiments publicly available and makes it difficult for those attempting to ensure animal welfare to challenge decisions made by the Home Office or actions by those holding licenses. This section of the ASPA was drafted prior to the FOIA and the result has been conflicts between the two Acts during attempts to gain information.

David Thomas, an animal lawyer and legal consultant to Cruelty Free International, has worked on animal experimentation cases extensively and highlights the issues faced in FOIA cases in this area. Thomas previously illustrated that ‘the outcome of some research is published, of course, but only if the researcher finds it advantageous to do so. He or she is unlikely to highlight the animal suffering involved. Negative results are rarely published. As a result, duplication is rife, as international institutions and the industry itself now acknowledge’ [49] (p. 198). Thus, the secrecy surrounding animal experimentation and the difficulties in accessing information can impact on assessing whether the harm–benefit assessment has been carried out appropriately, the necessity of the experiment and whether there are reliable results which can avoid future similar procedures. Even where results are published, they may not always be accurate. For example, factors such as funding from drug companies can impact on the reliability and bias of findings favorable to the product produced [50].

In FOIA requests concerning animal experimentation, the most commonly used exemptions to releasing information are found in ss.38 and 43 of the Act. S.38 exempts disclosure of information which would, or be likely to, endanger the physical or mental health of an individual or the safety of an individual. S.43 exempts disclosure where the information constitutes a trade secret. It is clear how these sections can be used to prevent disclosing information regarding animal experimentation, specifically arguments surrounding the endangerment to researchers from animal activist groups and the prejudice to commercial interests. These arguments arose in the case of the *BUAV v Newcastle University* (2011) [51], whereby the British Union for the Abolition of Vivisection (BUAV: Now Cruelty Free International) made a FOIA request for information of two project licenses which had authorized experiments on non-human primates. Newcastle University argued that the licenses were held by the named veterinarian surgeon on the establishment certificate, not by the university, who had to carry out personal duties under the ASPA and was exempt from disclosing the licenses under s.44 of the FOIA and thus s.24 of the ASPA. They also argued exemptions under ss.38 and 43 of the FOIA, which were not considered by the Information Commissioner in the BUAV’s complaint, as s.24 of the ASPA was enough. On appeal, it was held that s.24 of the ASPA did not apply, as the university had no ASPA functions and they could not, therefore, rely on s.44 of the FOIA. Again, the exemptions were not discussed under ss.38 and 43 of the FOIA and it was sent back to First-Tier Tribunal to consider. It was held that the exemptions under ss.38 and 43 of the FOIA did not wholly apply and it was ordered that the licenses be disclosed, subject to some necessary redactions where these sections did apply [52]. In total, Newcastle University spent £250,000 in legal fees resisting the FOIA case over a period of three years. In written evidence to the Justice Committee on their post-legislative scrutiny of the FOIA, Newcastle University complained of having to go through a lengthy and expensive procedure [53]. The BUAV responded stating that Newcastle was not forced, but chose to ‘run up astronomical legal fees’ and was ‘therefore the author of its own misfortune’ [54]. There has been much controversy surrounding such cases and the disputes continue. What is clear is that there is tension between the FOIA and the ASPA, and the ultimate issue of ensuring transparency in animal experimentation is still not yet achieved. More recently, Cruelty Free International faced similar difficulties gaining information from Imperial College London, who again relied on s.38 of the FOIA to refuse disclosing information relating to animals in experiments. Thomas, while appreciating the importance of s.38, opines that ‘it is all too easy for public bodies operating in controversial areas such as animal experiments to claim concern about safety and refuse to disclose information for that reason’ [55] (pp. 34–35). It can be difficult to gain necessary information to analyze whether licenses needed to be granted and, if so, whether the conditions of them and other elements of the ASPA were followed. The lack of transparency makes it difficult to confirm whether the ASPA is operating as intended and to identify further reforms or instances of good practice.

The Directive introduced a requirement of data sharing between Member States. Article 13 provided that procedures must not be applied if the data were already available in another Member State, obtained by procedures which are recognized by the EU regulatory requirements. This was in an attempt to ensure that studies are not duplicated, to be achieved through publications and presentations at conferences/scientific meetings. On a domestic level, the Government began to work with Research Councils and the NC3Rs to improve data sharing, including any negative results from procedures [56]. Further, Article 43 of the Directive requires anonymized non-technical summaries to be published, ‘subject to safeguarding intellectual property and confidential information.’ While exemptions similar to those faced under the FOIA are present, there is an attempt through the Directive to increase transparency. The summaries include information on the objectives of the project, including the harm–benefit assessment and the number and types of animals used, and the demonstration of compliance with the 3Rs [57]. Again, this activity was already happening in the UK, where approximately 400 abstracts a year were being published under a non-statutory voluntary scheme [58]. On the face of it, this Article looks promising, increasing the transparency of research and holding those with licenses accountable to their compliance with promoting the 3Rs and harm–benefit assessments, making it a statutory requirement. Also contained in the Directive, however, is a waiver of non-technical summaries for under Article 37(2), which Member States could include in their domestic law. It applies to categories of projects which ‘simplified administrative procedures may apply.’ This includes procedures which are classified as non-recovery, mild or moderate, not involving non-human primates and falling into specified categories of work. It was clear in responses to the consultation that many animal welfare groups did not want Article 37(2) to be used within the UK and the Government made it clear that they had no plans to use it, but transposed Article 43 as it stood. They committed to their intention for publication of non-technical summaries for all projects, stating it demonstrated their ‘continued, strong commitment to transparency’ [59]. It has been highlighted that the Directive’s intention of improved transparency has had little effect on the 3Rs within the EU, as the numbers of animals used in research has not decreased [60]. For example, research from Sweden reviewing applications for projects using animals in procedures found they were mainly not completed adequately and in line with the Directive. As for the project evaluation by the Swedish Animal Ethics Committees, only 4 out of 18 application reviews were deemed to satisfactorily mention harm and benefit and none included mention of the 3Rs [25].

As stated above, s.24 of the ASPA stops the Home Secretary from releasing any information received in confidence under the ASPA or obtained when discharging functions under the ASPA. This has made it difficult to contain information regarding animals used in experiments and decreased transparency within the UK. Since the Directive was transposed, the Government has looked at whether to repeal s.24 of the ASPA, due to the conflict with the spirit of the Directive of increased transparency. In 2014, a consultation was released, seeking views on retaining, repealing but creating new criminal offence or prohibitions on the disclosure of information about the use of animals in scientific research, or repealing s.24 altogether [61]. The preferred option of the Home Office was to repeal s.24 but with an amended legislative framework which would include a statutory prohibition on disclosing information relating to people, places and intellectual property. This would protect those involved in animal experimentation and any commercial rights, but allow for greater transparency. It was hoped that amending s.24 would ‘provide a constructive dissemination of technical knowledge’, which in turn could minimize the duplications of experiments involving animals [61] (p. 7). There was much acceptance of this consultation, and many came out in support of repealing s.24, with or without a new legislative framework. For example, The Academy of Medical Sciences tentatively supported all suggestions apart from retaining s.24, stating that the repealing of it would increase openness and transparency of the regulatory aspects of animal experimentation [62]. The National Anti-Vivisection Society noted that the FOIA now provided the necessary protection, meaning that s.24 is now obsolete [63]. This was supported by Balls, who highlighted that the FOIA works well in many other situations and should apply equally to the use of animals in experiments [64]. Unfortunately, the Government never released a response to the consultation and there has been no further progress on this issue. In 2017, Nature Watch commented on the need for s.24 to be repealed and the risk of the consultation being ‘shelved’ if no further action was taken [65]. This plea seems to have gone unheard, and the recognition of needing to reform this element of the ASPA since 1999 is again unactioned.

## 3. The Advantages of EU Action on Animals Used in Experiments

There have been many significant advances on the protection of animals used in experiments across Member States as a result of EU legislation, which was mainly inspired by the UK. The UK has banned cosmetic testing on animals since 1998. In 2009, the EU prohibited the testing of finished cosmetic products and cosmetic ingredients on animals [66]. The EU in 2013 prohibited the sale of cosmetics which have been tested on animals anywhere in the world, applying to both the final cosmetic product and the ingredients, irrespective of whether there are alternatives, often referred to as the ‘marketing ban’. Thus, cosmetic companies may still test their products and/or ingredients on animals, but it must be done outside of the EU and any results cannot be relied upon to sell the products in the EU. The UK also banned licensing of research and testing on great apes in 1997, which was followed by the EU in the Directive, subject to a safeguard clause set out in Article 55(2). It appears that the EU has often followed the UK’s lead in terms of animal welfare, mirroring the legislation for animals used in experiments, and the UK has achieved animal welfare goals much earlier than the EU [67]. There are, however, other advantages gained, beyond the Directive, from being a Member State of the EU and advancing the protection for animals used in experiments, which will be outlined below.

### 3.1. REACH

Regulation (EC) No. 1907/2006 concerning the Regulation, Evaluation, Authorisation and Restriction of Chemicals (REACH) applies to all substances manufactured or imported into the EU of one ton or more. It came into force in 2007, and it is overseen by the European Chemicals Agency (ECHA) with the aim of protecting human health and the environment from the risks that can be caused by chemicals. Further, it promotes the 3Rs to reduce the number of tests needed on animals. It applies to all chemical substances, including those found in electrical appliances and cleaning products, collecting and assessing information of the substances, their properties and hazards. Responsibility is placed on the industry, with manufacturers and importers having to gather the information relating to the properties and hazards and register this in the ECHA’s central database.

The benefit of REACH is the data sharing which comes from the central database, and the ECHA highlight that ‘Registrants may only carry out new tests when they have exhausted all other relevant and available data sources’ [68]. The ECHA require that companies producing or importing the same substance must share information about the properties of their substance, and companies registering the same substance must jointly submit any results of testing on vertebrate animals. The ECHA is clear that any reliable studies on animals must not be repeated, encouraging companies to use other methods first. They organize training sessions on alternative methods, publish advice and a large amount of data from registration on their website, and has made available software for companies to identify data which may be relevant when assessing their own substances. The overriding aim, though, is the protection of human health and the environment under REACH, and the promotion of non-animal approaches will not ‘undermine’ this [69].

As a result of Brexit, the UK has lost its seat on the ECHA Member State Committee and access to the EU REACH database, potentially increasing the risk of duplicate experiments on animals, which could ‘slow down progress’ of the development on non-animal alternative methods [70]. There is no alternative to the ECHA in the UK and McCulloch has highlighted that Brexit will impact greatly on animals used in experiments, which may ultimately ‘lead to the unnecessary testing on animals’ [71]. This was discussed during oral evidence given to the Energy and Environment Sub-Committee of the House of Lords: Select Committee on the European Union, on the future of REACH. It was highlighted that continuing access to the database was the best outcome for animals used in experimentation. It was acknowledged, however, that this may not be negotiated and that the UK will have to take a regulatory approach in this situation. Thus, ‘if that required animal testing, it would require animal testing’ [72]. Without a domestic alternative to REACH in the UK, a replacement has been brought into domestic law called UK REACH [73], retaining the key principles of EU REACH. UK REACH ‘grandfathered’ the EU REACH registrations submitted by UK companies prior to exit day where UK Registrants owned the test data. Where they did not own the test data, difficulties could be caused in trying to source it to add to UK REACH and EU owners of the data are under no obligation to share it with UK registrants. There was no negotiated provision in the Trade Cooperation Agreement for the UK to access the EU REACH database and, though discussed in Parliament, no suitable provisions for avoiding duplicate tests on animals has been put forward yet [74]. It is important to note that EU REACH is still in operation in Northern Ireland. This means the UK now have two systems in operation and those supplying or purchasing substances to the EU need to meet the relevant duties under both systems. Instead of the ECHA exercising regulatory functions under the legislation, the Health and Safety Executive (HSE) now carry out this role in Great Britain.

Further developments on REACH have been discussed during the Environment Bill 2020 [75], with frustrations expressed at the lack of provisions for animal welfare and the opportunity to decrease the number of animals used in chemical testing [76]. The Environment Bill has been carried over into the next Parliamentary session and has just completed the report stage in the House of Commons at the time of writing [77]. After concerns of UK REACH and the impact on animal testing, there has been a new clause added to provide for this. The clause states that the Secretary of State must set targets for the replacement of tests on animals within the scope of REACH and the reduction pending replacement of the number of animals used and the suffering they endure [78].

It is too early to tell whether the loss of access to the EU REACH database has had an impact on the number of experiments conducted on animals, and close monitoring over the next few years will provide us with evidence of whether these concerns have been realized. It must be acknowledged that research using animal models is expensive and time-consuming meaning duplicate studies are not desirable for companies, in addition to the ethical reasons.

### 3.2. Advancing the 3Rs

As stated above, the Directive brought the 3Rs into law, but the UK has been somewhat slow to fully get on board with them. When considering the lack of a reduction in the number of animals used in experiments, Sparks and Brooman highlight that the ‘UK is a chief culprit in failing to reduce the number of animals used’ in experiments [67] (p. 10). Though, as highlighted in the introduction, the UK are often said to be a world leader in animal protection, we subject more animals to scientific procedures than any other Member State within the EU. The latest report published by the EU in 2020 provides statistics from the period 2015–2017 on the total amount of experiments carried out on animals across the Member States, presented in Table 2 below. The table also contains the number of total experiments carried out on notable species:

In 2017, 9.39 million animals were used across Member States, of which 61% were mice, with dogs, cats and non-human primates making up 0.3%. During the period 2015–2017 there was a decrease in the number of dogs and cats used, but an increase in the use on non-human primates by 15% [27].

In 2017, the UK used the highest number of animals, followed by Germany and France. Together, these countries made up 60% of uses in 2017 [79]. To show the use of animals over the EU reporting period, Table 3 displays the total amount of animals used of selected countries for comparison: 

It is important to note that the numbers provided to the EU by the UK are lower than what is published by the Home Office in the national report. This is because the UK publish all procedures, including those in the sub-threshold severity category, which the EU do not require, for example. The actual total amount of animals used in procedures in the UK during 2015 was 4.14 million, 3.94 million in 2016 and 3.79 million in 2017, which is significantly more than displayed in Table 3. For consistency between Member State figures, figures published in the EU report using their reporting system are used for comparison. From these figures, it is not clear why the UK has not significantly reduced the number of animals used in experiments, but it has made it clear that their figures include the number of genetically altered animals, which some other countries like Ireland and Denmark do not include [2]. The other Member States presented above, however, have also not significantly reduced the number of animals used in experiments, and arguably the UK has done better at using less animals. As can be seen from Table 3, the number of animals uses in experiments in the UK has decreased since the transposition of the Directive, but had remained the Member State using the highest number of animals in experiments and significantly more than some other Member States. The Home Office attributes the recent rise of experiments to an increase in breeding genetically altered animals, which rose by 43% between 2007 and 2019, the period of reliable data [2]. It can be argued that the advancement of the 3Rs in the Directive has had little impact in the UK when looking at the figures, and a simple insertion of their definition and use into the ASPA has not done what the Directive intended. It also appears to have had little impact in other Member States. What is at risk, however, is the sharing of practices between Member States in how to reduce the number of animals used in experiments and collaboration on finding alternative non-animal methodologies, which may be lost with Brexit. For example, the European Parliament in 2020 voted to replace chemical testing with non-animal alternatives in their EU Chemicals Strategy for Sustainability. They recognize the need to innovate chemical testing methods, to reduce the dependency on animals testing and improve the quality and speed of chemical testing [80]. This will include revisiting EU REACH with potential revisions to achieve their objectives, which the UK will not be party to.

A concern raised as a result of Brexit was continued access to research funding from the EU, specifically the Horizon 2020 program. It was negotiated that those who had ongoing projects which are due to finish after 1 January 2021 would continue to receive the EU funding for the lifetime of the project, but it was unclear what the position would be after this time. It is imperative that the UK maintains access to such funding, as it can help advance non-animal testing technologies and help further promote the 3Rs. Over the period 2012–2016, the EU reported the funding of 69 research projects mainly focused on alternatives to animal models [41] (p. 105). For example, EU-ToxRisk is a project mainly funded through Horizon 2020, of which the EU contribute €27,798,299, and has UK participants. The aim of this integrated European program is to ‘drive a paradigm shift in toxicology’ towards animal-free chemical safety assessment. They highlight the current issues in reliability and ethics of using animals in assessing chemical safety and the urgent need for modern and reliable approach, with this program pushing toxicology towards ‘mechanistic animal-free safety assessment’ [81]. Programmes such as this are clearly advantageous to be a part of, with the UK not only helping develop non-animal models, but hopefully to be influenced to decrease the number of animals used domestically in experiments. It has now been announced that the UK will associate to Horizon Europe running from 2021 to 2027, subject to paying a financial contribution, providing access to funding under the program on equivalent terms as organizations in EU countries [82]. This result of Brexit negotiations is arguably a positive outcome for animals used in experiments, with the ability to continue working with European partners in advancing non-animal models.

## 4. What Next for the UK?

While the UK has found itself in some detrimental positions in terms of the welfare of animals used in experiments, there are also some opportunities having left the EU. The UK have the chance to enhance the protection of animals used in experiments and provide further support for alternatives and advancing the 3Rs. Without the restraints of Article 2 of the Directive, and in some respect membership of the EU generally, the UK now have freedom to become a world leader in laboratory animal welfare again. There is also, however, the risk of UK laws becoming weaker, or staying as they are. Since the referendum, there have been many concerns expressed about the risks to animal welfare laws and that welfare could be compromised in favor of other concerns, such as trade [83]. These concerns have been vocal, and while the Government have been exploring trade deals, particularly with regard to farmed animals, they have been influenced by stakeholders and the general public and their fears of lower animal welfare standards [84]. The current Conservative Government have been exploring strengthening animal welfare laws and have launched a ‘flagship Action Plan’ discussed in the introduction [85]. The improvements announced include introducing laws to tackle puppy smuggling, make it illegal to keep primates as pets and the ban the import of hunting trophies. It relates to many different kinds of animals, from companion, to farmed, to wildlife, but animals used in experiments do not appear anywhere. This lack of focus is disappointing, and may result in the current standards being maintained or weakened. The current Government seem to be focusing on animals used in experiments outside of legislation, in the attempt to become a leader in non-animal models, discussed below. This section of this article does not address the risks of Brexit, as they have been written about elsewhere [86], but instead focuses on the opportunities leaving the EU can bring and how the UK Government may seek to improve the ASPA.

### 4.1. Strengthening the ASPA and Domestic Legislation beyond the Directive

As stated in the Section 2 of this article, Article 2 of the Directive imposed limitations on Member States’ ability to provide greater protection than afforded in the Directive, save for where that protection was already in place. Arguably, leaving the EU allows the UK to strengthen animal welfare laws, including laws governing animal experimentation, and may no longer be ‘confined by the compromise of agreements between many nations’ [67] (p. 10). As a minimum, the Government have announced plans to retain aspects of EU law which protect animals in experiments, most notably in cosmetic testing. There was a great worry that any potential trade deals would allow for products to enter the UK market which had been, or contained ingredients which had been, tested on animals [14]. The Government’s commitment to the ban is very much a relief for those concerned with animal welfare. They could, however, go further than the law provided by the EU, such as banning the testing of Botox on animals, which currently falls outside of the Cosmetics Regulations though arguably should be banned when considering the harm–benefit assessment [87].

There is also much impetus for removing the category of ‘severe’ suffering. The RSPCA has been advocating for this for some time [88], organizing meetings to discuss reducing or avoiding experimental procedures and leading on the generating of guidance [89]. This is an area which can now gain greater traction without the restraint of Article 2, and the RSPCA have announced their intention to campaign for the end to severe suffering in animal experiments in their 2021–2030 strategy [90]. Whereas previous developments were for a policy change to not license procedures which involve severe suffering, leaving the EU means that there can be an actual legislative change to end this practice and increase welfare for animals used in experiments.

### 4.2. Advancing the 3Rs

The RSPCA highlight that there is a significant amount of funding from the EU for research, which includes scientific research conducted on animals. As highlighted in the previous section, UK researchers have been worried about the loss of access to this funding and the Government has promised further domestic funding for research. Prior to Brexit, Innovate UK claimed that the UK could become a leader in non-animal technologies, with a result of emerging technologies and industries ‘driving future economic growth’ [91]. Further, it was noted above that there is a lack of legislation and policy driving the 3Rs, which would provide support to the scientific community to develop further non-animal methods, reduce the number of animals used and potentially improve the reliability and quality of the research. It is imperative, therefore, that the Government continue to provide funding, but increase the financial support and initiatives for using and developing non-animal methodologies, rather than continued substantial use of animals in experiments. The Government may, for example, want to take inspiration from NCad’s strategy on how to proceed with this. They could use the Delivery Plan from 2014, update it, and provide clear timeframes and numerical goals to advance us towards a reduction in the number of animals used in research, rather than what appears to be little more than a wish list. There have also been recommendations of revisiting the 3Rs, so that they are not only about animal welfare, but also scientific and social value. Stretch and Dirnagl, for example, argue for the need of an additional 3Rs on scientific value, Robustness, Registration and Reporting, to be taken into consideration during planning, licensing, and funding of experiments using animals [92]. They acknowledge that the NC3Rs is already doing this through guidelines in some aspects, but they need to be more strongly integrated as a set of ethical principles.

There are some positive changes coming in the future of animals used in experimentation in the UK. An all-Party Parliamentary Group, the Human Relevant Science Parliamentary Group, was formed in 2020, bringing together Lords, Members of Parliament and other third sector groups and stakeholders, supported by Alliance for Human Relevant Science. Their aim is to ‘accelerate the development and uptake of human relevant life sciences in the UK.’ The strategy to do this is by replacing poorly performing animal tests with human relevant methodologies, arguably innovative and new in their approach and increasing the efficiency and profitability of the industries involved [93]. In order to do this, they have a list of objectives, mainly focused around exploring the current Government financial support and funding opportunities and what financial support is needed to advance New Approach Methodologies, as well as examining the current regulatory framework and make proposals for changes [94]. The purpose of human-relevant research is to promote human-focused methods, due to the issues associated with animal use in research. Lees argues that the new group ‘provides a great opportunity to drive positive progress towards the use of alternatives to animals in research, alongside the prospect of new, improved and possibly safer medicines as a result’ [95] (p. 18). This has also been welcomed by relevant NGOs, such as Cruelty Free International [96]. In light of losing access to the EU REACH database and the UK seat on the ECHA, it can only be positive that the Government is becoming more serious about non-animal methods, for the benefit of human and nonhuman animals alike. This is also in line with recommendations from the results of the Animal Protection Index, who suggested that the UK Government created a multi-stakeholder committee dedicated to developing alternatives to animal methodologies [87].

### 4.3. Advancing Transparency

As outlined in this article, while transparency has been provided for in domestic legislation, it can be limited and difficult to access information on project licenses. NGOs argue for greater transparency of experiments involving live animals, including making project license applications and retrospective reviews available publicly [14]. This will be helped by repealing s.24 of the ASPA and removing the prohibition on the Home Office of sharing information, such as license applications and inspection reports. Releasing this information, in a way which provides for safety of scientists and their commercial interests, will provide opportunities to assess whether experiments on animals had proper consideration of the harm–benefit assessment and whether the 3Rs were fully considered. This is turn will help ensure testing is only done when necessary and with an actual need, while also instilling more public trust in the system. Again, this was a recommendation from the Animal Protection Index, criticizing s.24 for essentially operating as a ban on the releasing of information regarding animal experimentation [87].

Moreover, transparency links into better public education, more understanding of procedures and less perceptions of secrecy [97]. Although not highlighted throughout this article, there is arguably a need for better public education on animals used in scientific procedures which must be briefly highlighted. Research carried out by Ipsos in 2018 found that 38% of the public still believe that the testing of cosmetics on animals is allowed in the UK, though banned by the UK in 1998 and the EU in 2009, which was an increase from 31% in 2014. During this time, there has been an increase in the public wanting to learn more about alternatives to animals used in research and, though public acceptance of the use of animals in research has remained relatively the same, the proportion of the public who are not concerned with the use of animals in research has fallen to 15% from 22% in 2016 [98]. It is clear that the public are not well informed on animals used in scientific procedures and, more importantly, there is a general want to be. The Government should be taking advantage of this, providing opportunities and learning resources. This in turn will help to achieve better welfare conditions for animals and public engagement in debates.

## 5. Conclusions

Though the EU have been instrumental in improving welfare standards across Member States, it is arguable that the UK were ahead by domestically introducing their reforms first and influencing EU policy and legislation. As highlighted in this article, much of the EU legislation has been based on already existing UK legislation, with some minor changes being implemented. What was beneficial of EU legislation, such as the Directive, was bringing other Member States up to the minimum standards expected for the welfare of animals used in experiments, particularly in areas such as severity limits, transparency and the advancement of the 3Rs. Unfortunately, however, the Directive introduced restrictions under Article 2 on Member States of going beyond the minimum protection and shattered the illusion of the furtherance of animal welfare with ‘safeguard’ clauses, such as Article 55. It has been an advancement for animal welfare in some respects, but there have also been ‘missed opportunities’ and issues with implementation, as discussed in this article. In the short term, it seems that Brexit may be a curse for animals used in experiments in the UK. While the UK will retain EU law within domestic legislation, we have lost access to the EU Reach database. This means there is a risk of duplicate experiments on animals needed in order continue marketing chemicals. The other worries which were born from Brexit, however, seem to have resolved themselves, such as the Government committing to keeping the cosmetic testing ban and researchers still able to access funding through the Horizon Europe program.

Exploring the long-term implications, the opportunities that arise from Brexit could allow the UK to become a world leader again in the welfare of animals used in experiments, if the UK choose to take them. The UK have dopped in the Animal Welfare Index, scoring particularly low for the protection of animals used in scientific research. Highlighted in this article are ways the UK can strive towards better protection and move from a B to A overall. The UK can become a leader in the replacement of animals used in experiments, developing more non-animal models where possible. This has already been identified as a growing economy within the UK, with the opportunity to develop innovative non-animal methodologies. This in turn will further develop and embed the 3Rs into the legal system and scientific communities, working towards the ultimate goal of replacing animals in scientific procedures. In order to do this, however, the Government needs to prioritize funding in this area and provide training and support within scientific communities for the use of non-animal methodologies. The NC3Rs is doing this on a great scale already, but more support is needed. Without the restraints of EU legislation, particularly that of the Directive, the UK can increase the welfare provided for animals used in experiments, particularly reviewing severity limits and reducing procedures which are in the severe category. The UK can also increase transparency of animal experimentation, by repealing s.24 of the ASPA and ensuring that there is greater education of the public on the use of animals in research. The EU has been a positive influence on the welfare of animals used in experiments across Member States, but it is again time for the UK to move forward in animal welfare standards and become a world leader once more.

## Figures and Tables

**Table 1:** Severity Classifications of Licenses Granted in EU 2017 [27] (p. 10).

Severity Classification	Number of Licenses Granted
Non-Recovery	621,054 (6%)
Mild	4,865,721 (51%)
Moderate	3,071,828 (32%)
Severe	1,023,138 (11%)

**Table 2:** Total Number of Animals Used in Experiments in the EU in the Period 2015–2017 [27].

Year	2015	2016	2017
Total number of animals used	9,590,379	9,817,964	9,388,162
Total number of mice	5,711,612	5,989,413	5,707,471
Total number of rats	1,201,189	1,173,135	1,146,299
Total number of dogs	14,501	15,691	13,688
Total number of cats	1975	1951	1879
Total number of non-human primates	7136	7239	8235

**Table 3:** Comparison of Total Number of Animals Used in Experiments across Selected Member States [41].

	2015	2016	2017
UK	3,168,480 (1.07 million related to the creation/breeding of genetically altered animals)	2,790,392 (751,000 related to the creation/breeding of genetically altered animals)	2,574,875 (669,000 related to the creation/breeding of genetically altered animals)
Germany	2,045,261	2,128,254	2,068,813
France	1,901,542	1,918,402	1,914,174
Ireland	228,339	226,934	242,302
Netherlands	479,580	386,700	477,550
Denmark	243,792	289,225	237,949
Czech Republic	229,869	237,662	241,712
Poland	176,980	188,719	156,234
Portugal	20,623	31,712	52,370
